# Median Sternotomy for Mediastinal Mass Removal: Thymoma of Unknown Cause

**DOI:** 10.7759/cureus.88364

**Published:** 2025-07-20

**Authors:** Kaitlin N Murphy, Arian Hoodfar, Mitchell Katkic, Cemil Purut

**Affiliations:** 1 Internal Medicine, Edward Via College of Osteopathic Medicine (VCOM), Roanoke, USA; 2 Research, Edward Via College of Osteopathic Medicine (VCOM), Blacksburg, USA; 3 Orthopedic Surgery, Edward Via College of Osteopathic Medicine (VCOM), Blacksburg, USA; 4 Cardiothoracic Surgery, Edward Via College of Osteopathic Medicine (VCOM), Salem, USA

**Keywords:** incidental tumor, median sternotomy, mediastinal tumor, myasthenia gravis, thymoma

## Abstract

Thymoma is a rare mediastinal malignancy. Although it may often be associated with paraneoplastic syndromes such as hypogammaglobulinemia, pure red cell aplasia, or myasthenia gravis (MG), some thymomas may also be idiopathic in origin. Although thymoma is the most common mass found in the anterior mediastinum, its rarity still proves difficult in swift diagnosis and subsequent treatment. This case presents a patient with an unknown anterior mediastinal mass incidentally found on imaging, with the final diagnosis of thymoma made from a pathologic investigation that was performed post-sternotomy and resection.

## Introduction

Although thymoma is the most common anterior mediastinal tumor, it has an overall estimated incidence of 0.13 in 100,000 people [1]. Thymoma can be found in all age groups. However, the largest peak appears to be in those between the ages of 30-75, and there does not seem to be a gender predilection [2]. The imaging modality of choice for evaluating a thymoma is computed tomography (CT), as this can help clinicians distinguish between thymoma and other anterior mediastinal masses [3]. Thymoma can be differentiated into various types, and Masaoka staging is most commonly used for this evaluation, with stage IV having the worst prognosis and highest risk of recurrence [4]. Earlier-stage thymomas have a lower rate of recurrence and a higher survival rate. The Muller-Hermelink morphologic classification can also be used to determine encapsulation versus invasion of surrounding tissues by the thymoma [5]. Additionally, studies have found that total thymectomy should be recommended to patients with an early Masaoka stage, as this will greatly improve clinical outcomes [6].

Earlier clinical stages, such as stage I and II, are the histopathological subtypes most often seen in patients with myasthenia gravis (MG) [3]. Ten-year survival rates for stage I and II thymomas are 90% and 70%, respectively [5]. Because of the close association with MG, neurological symptoms such as weakness or diplopia are most often the presenting symptoms for patients with early-stage thymoma. However, some patients may be asymptomatic or present with chest pain or tightness, cough, or other nonspecific symptoms. Interestingly, one study that reviewed 241 cases of thymoma demonstrated that 82.4% of stage II thymomas were associated with myasthenia gravis [4,7].

While thymoma is typically associated with myasthenia gravis, it may also arise as a part of other paraneoplastic syndromes (PNS). A recent study found that 123 paraneoplastic syndromes were associated with thymoma, with the five most common being myasthenia gravis, pure red cell aplasia, lichen planus, Good's syndrome, and limbic encephalitis [8]. Other studies have also found associations with acquired neuromyotonia, myositis, and Morvan's syndrome [9,10]. As with the studies on MG, removal of the thymoma in patients with these paraneoplastic syndromes has also shown resolution in their symptoms related to the PNS. While some type of syndrome or disease process commonly accompanies or even influences the discovery of a thymoma, these tumors can rarely appear without the presence of one of these comorbid conditions.

## Case presentation

**Figure 1:**
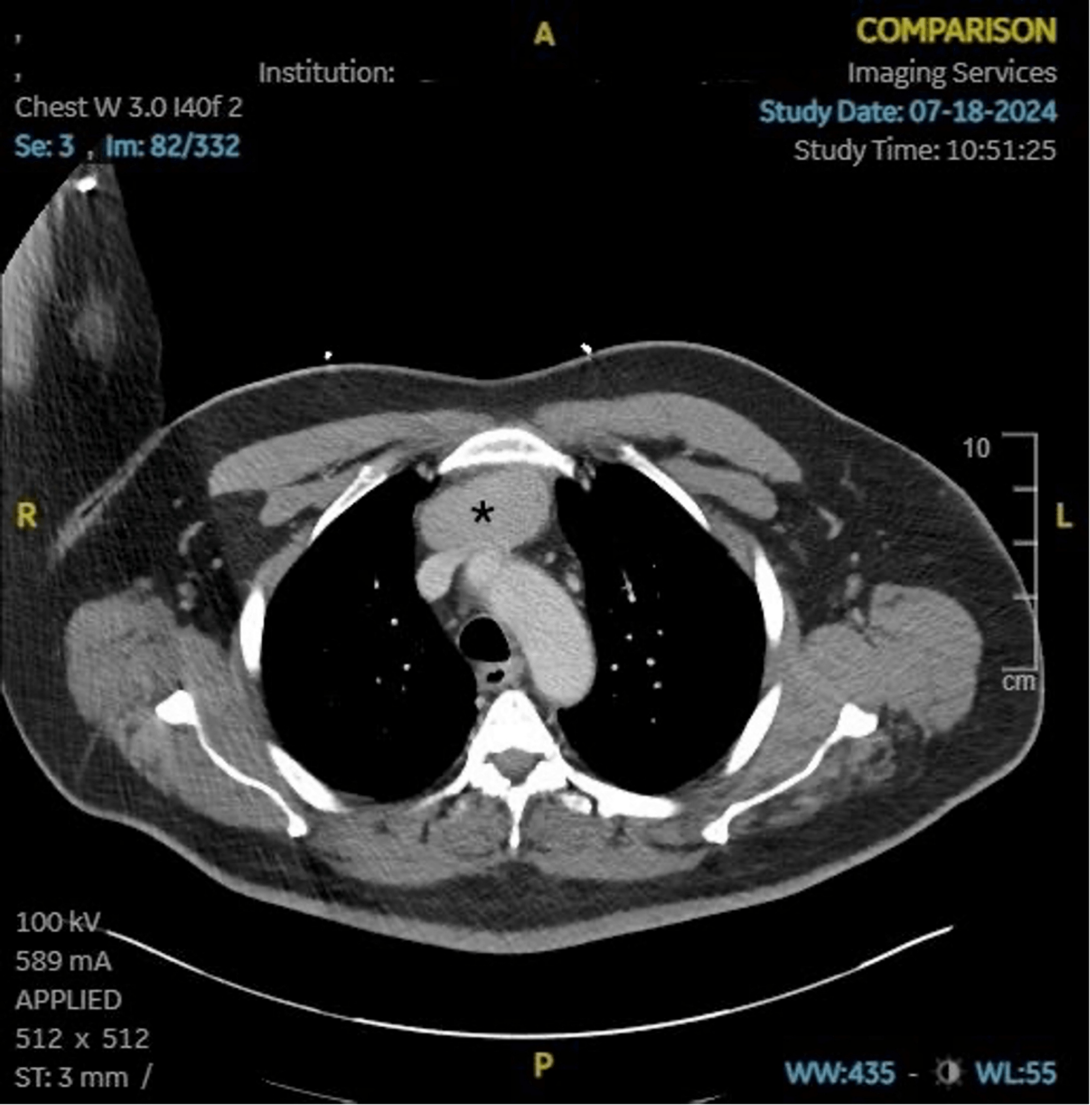
Preoperative CT chest with contrast * indicates mediastinal mass

This patient is a 61-year-old Caucasian male with a past medical history of cerebrovascular accident (CVA), hypertension, hyperlipidemia, type II diabetes mellitus, gastroesophageal reflux disease (GERD), and migraine with aura who was referred to cardiothoracic surgery for the presence of an anterior mediastinal mass of unknown origin on CT imaging. The mass was incidentally found on prior CT imaging of the chest and neck when assessing for a right ocular stroke during a recent hospital admission. We presume that this CT was completed to rule out any carotid disease or apical lung disease that could contribute to ocular stroke symptoms. At the time of the stroke, the patient had transient blindness of the right eye, which self-resolved, and he did not suffer any debilitating neurological deficits. There was no palpable fullness on chest exam, no voice changes, or venous engorgement, which may be seen in other patients with mediastinal masses. Upon review of the CT chest imaging, there was a lobulated, soft tissue density mass measuring 8.3 x 3.6 x 5.7 cm in the anterior mediastinum (Figure 1). 

The differential diagnosis included but was not limited to thymoma, lymphoma, germ cell tumors, teratoma, and benign thyroid mass. The patient was given the ability to explore options for management, including minimally invasive robotic surgery at a different hospital, or a sternotomy and thymectomy with us. Shared decision making and extensive discussion were held, and the patient ultimately decided to undergo the open procedure and thymectomy. 

**Figure 2:**
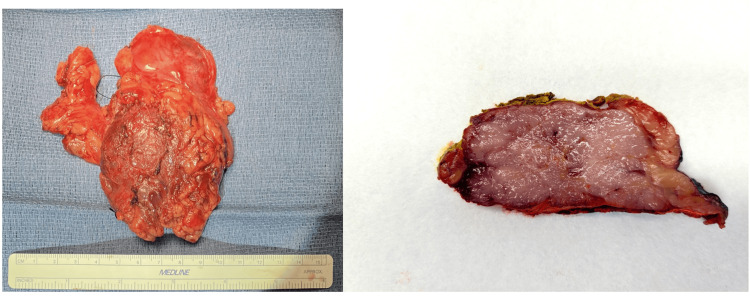
Left) Gross specimen of mediastinal mass measuring 10.5 x 9.2 x 3.5 cm mediastinal mass; Right) Cross-sectional cut with a firm tan-white gritty cut surface with a slightly fish-scale surface surrounded by adipose tissue

At the time of admission for removal of the mass, approximately 45 days from the discovery of the initial CT findings, the patient exhibited no neurological symptoms and no evidence of MG. The patient underwent his scheduled median sternotomy, allowing for complete resection of the 10.5 x 9.2 x 3.5 cm mediastinal mass (Figure 2), which was then sent off to pathology for further analysis. Pathology report of the mediastinal mass was significant for a thymoma, type B2 stage pT1a pNx pMx per the American Joint Committee on Cancer (AJCC) Eighth edition staging system and stage 2b per the modified Masaoka staging system with macroscopic capsular invasion into thymic or perithymic fat, or grossly adherent to, but not breaking through, mediastinal pleura or pericardium. This patient's mass fits this description as there was no invasion into the pleural space, and the surrounding tissue was described as "hemorrhagic adipose" per the pathology report. The patient was made aware of the findings and referred to radiation oncology for adjuvant radiation therapy. No further surgical intervention was performed. 

**Figure 3:**
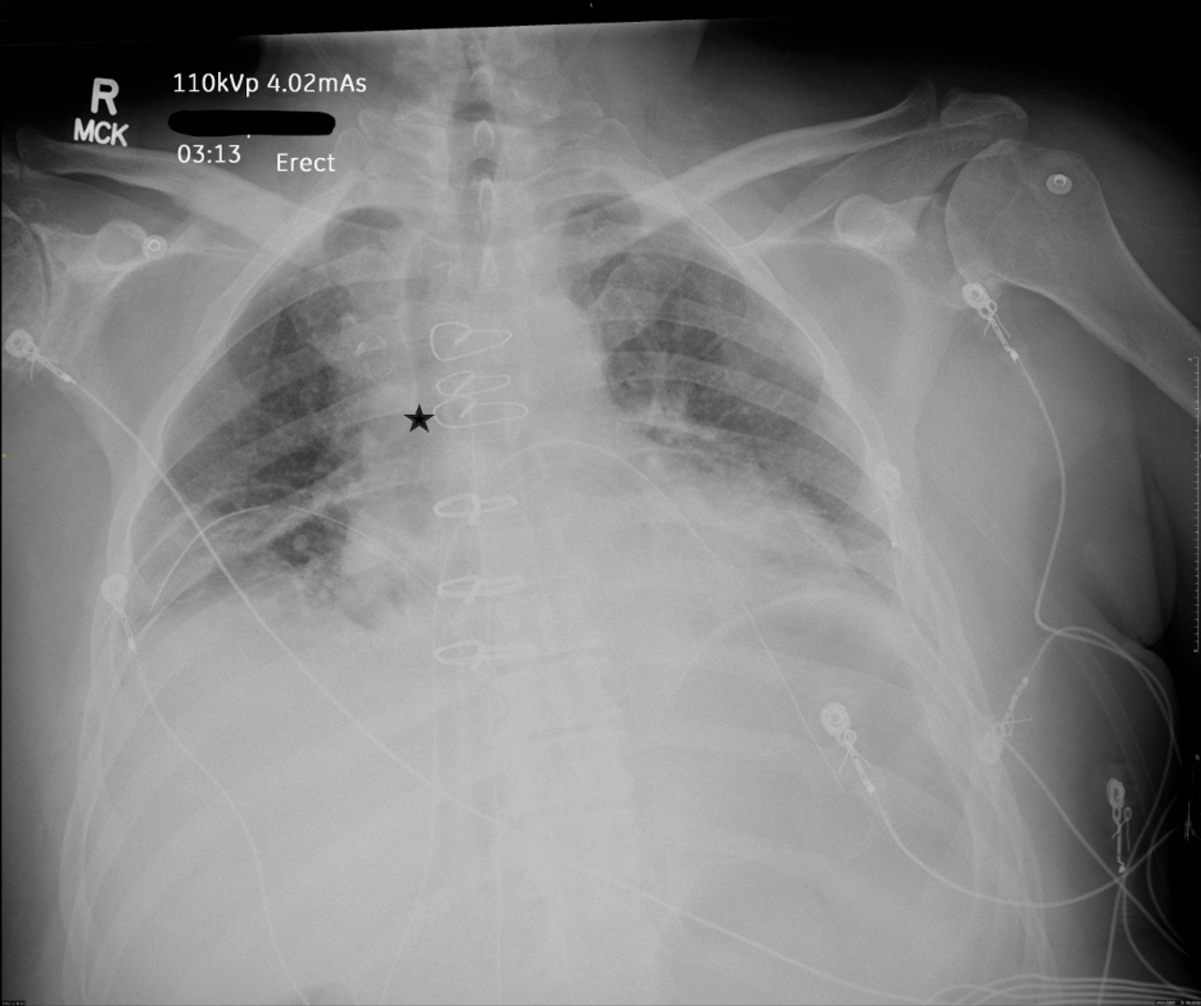
Post-operative chest X-ray on post-operative day one demonstrating six sternotomy wires after thymectomy, one of which is denoted by the star

On post-operative day one, the patient reported sternal soreness, dyspnea on exertion, and difficulty with deep inhalation. Physical exam showed coarse crackles and decreased breath sounds, maintained on 2 liters of oxygen via nasal cannula. Chest X-ray subsequently showed mild interstitial edema bilaterally and appropriate chest tube placement (Figure 3). Labs were notable for a white blood cell count of 20.61 (normal 4.5-11.0 x 10^9/L) with 77.7% neutrophils (normal 40-70%). Throughout the hospital course, the patient also struggled with hyperglycemia with glucose values between 200-300 mg/dL (normal range 70-110 mg/dL), which was managed by inpatient endocrinology. The patient otherwise had a routine post-operative course with no major complications. Subsequently, his hyperglycemia improved with glucose values between 120-160 mg/dL, and his leukocytosis also improved to 12.88 with 60.5% neutrophils on post-operative day three. He was discharged on post-operative day three with wound care instructions and returned to follow up with the cardiovascular team as well as an outpatient visit with radiation oncology for further management. 

## Discussion

Because thymoma is a rare mediastinal mass, it is important to be able to delineate between the various processes that may be associated with this thoracic tumor. However, in rare cases, thymoma can be idiopathic and not seen with commonly associated diseases or paraneoplastic syndromes [8]. The patient's presentation of idiopathic stage 2b (Masaoka staging) thymoma is a rare example of the disease, as 82.4% of this subtype are associated with myasthenia gravis [7]. Our patient presents an interesting vignette as the histopathologic staging does not seem to correlate with his clinical presentation, suggesting he could have had underlying indolent disease or that this is simply a rare coincidence. It should be noted that this patient did not have myasthenia gravis testing completed during this particular hospital stay (antibodies such as anti-AChR, anti-MuSK), but it may have been done during an outpatient follow-up that our team was not a direct part of, and should be considered in any patient with a finding of a new thymoma. 

Suppose a thymoma is found incidentally on chest imaging. In that case, removal of the tumor is recommended, as various studies have demonstrated better outcomes with thymectomy rather than conservative measures [6]. This is significant, as this medical management may not be well-known given the rarity of mediastinal tumors and their occurrence. Computed tomography with intravenous contrast is the ideal imaging modality for initial diagnostic purposes, and fine needle aspiration, core needle biopsy, or even open biopsy is the most typical next step for further tissue diagnosis [8]. The Masaoka classification is used to stage the tumor, and many studies have found very positive prognoses regarding five and 10-year survival rates for completely resected thymomas [8].

This case report highlights the importance of prompt diagnosis and treatment of mediastinal masses, including thymoma, even when it is found incidentally and thought to be without relation to the patient’s clinical condition. Staging of a thymoma may help in understanding its related conditions, although some cases may not demonstrate any correlation to associated diseases. While these rare events can give rise to difficult medical decision-making for clinicians, this case highlights the importance of utilizing current medical guidelines for surgical cases and subsequent histopathological investigation to determine the correct diagnosis and course of action. 

## Conclusions

This patient's case demonstrates how thymoma can be found as an incidental mediastinal mass without underlying causes such as MG or paraneoplastic syndrome. Typically, patients who have a thymoma related to MG exhibit symptoms like diplopia, muscular weakness, and difficulty with daily tasks such as rising from a chair or climbing stairs. Since the patient did not exhibit any of these symptoms and serum antibody testing and/or electromyography (EMG) testing was not completed, it can be assumed he does not have MG. Because his histopathologic staining was consistent with a subtype of thymoma most often seen in MG patients, this is an interesting case that suggests more research is needed to understand the subtyping of idiopathic thymomas.
